# Supplementary material for: Enhanced stability and polyadenylation of select mRNAs support rapid thermogenesis in the brown fat of a hibernator
Source: eLife. 2015 Jan 27;4:e04517. doi: 10.7554/eLife.04517 (PMC4383249; doi:10.7554/eLife.04517)
Supplement: Supplementary file 2. — Mathematical Modeling of Transcript Dynamics. DOI: http://dx.doi.org/10.7554/eLife.04517.020 [file elife04517s002.docx]

**Supplementary file 2. Mathematical Modeling of Transcript Dynamics.**

To investigate RNA transcript dynamics across a torpor-arousal cycle, we developed a differential equations-based mathematical model simulating the abundance of 1400 bulk and 50 protected transcripts. This ratio of bulk Class I transcripts to protected Class II transcripts reflects the ratio observed in the data (14267 bulk; 531 protected in DIANA Clusters 5 and 6, Figure 2D). All modeling and model analysis were performed in MATLAB (MathWorks, Natick, MA). The abundance of each RNA transcript, *R*, was governed by the ordinary differential equation:

**
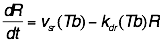
**

where v_sr_ is the rate of RNA synthesis, and RNA degradation is proportional to R with a degradation rate constant k_dr_. Both v_sr_ and k_dr_ are functions of body temperature, Tb. This characterization of RNA transcript dynamics has been described previously for fixed temperatures (Schwanhausser, Busse et al. 2011). To model RNA transcript dynamics under variable temperature conditions, we set values v_sr,w_ and k_dr,w_ for the warm animal according to established log-normal distributions for rates of transcription and half-lives, respectively (Schwanhausser, Busse et al. 2011) and adjusted them for other Body temperatures using Q10 effects. Synthesis rates in the warm animal v_sr,w_ (mRNAs/min) were drawn from a log-normal distribution with log mean mu = 0.0333 and log standard deviation sigma=0.015. Half-lives in the warm animal, t_1/2_ (h), were drawn from a log-normal distribution with log mean mu = 2.5 and log standard deviation sigma = 0.3. Half-lives t_1/2_ (h) were converted to degradation rates k_dr,w_ (mRNAs/min) as follows:


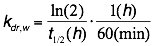


Temperature-dependence for transcription and degradation was established using Q10_T_=3 and Q10_D_=2, respectively (Burka 1969, van Breukelen and Martin 2002). Transcription (v_sr,w_) and degradation (k_dr,w_) rates for the warm animal were adjusted for Tb as follows:

$v_{sr}(Tb)={(v}_{sr,w})\cdot({Q10}_{T}^{-(37-Tb)/10}$)

$k_{dr}(Tb)={(k}_{dr,w})\cdot({Q10}_{D}^{-(37-Tb)/10}$)

During the torpor-arousal cycle, Tb undergoes a rapid transition from approximately 37°C to 5°C. We modeled this body temperature profile over 12 days from the end of an interbout arousal period when Tb is 37°C to late torpor when Tb has been at 5°C for over 10 days (Figure 5—figure supplement 1) and used this profile to drive the temperature-dependent RNA dynamics across the torpor-arousal cycle.

The differential equation governing the change in R specifies that steady states of R occur when R= v_sr_/k_dr_ (Figure 5—figure supplement 1). For each RNA transcript R, we set the initial condition R_0_ to be the steady state abundance in the warm animal: R_0_=v_sr,w_/k_dr,w_. The sum of steady state abundances across transcripts represented the total initial EDGE-tag count for our simulated transcript population. We simulated transcript abundance over the 12 day (17200 min) torpor-arousal cycle. Equations were solved numerically using the MATLAB built-in solver ode45 with relative error tolerance 1e-6.

The simulated abundance of RNA transcripts at day 12 represents the raw abundance of these transcripts in late torpor. To compare the abundances predicted by the model to patterns observed in EDGE-tag count data, we normalized the abundances from late torpor to maintain a fixed total EDGE-tag count across the simulation. For each parameter set, we ran 5 simulations and reported the mean effect on the relative abundance of protected transcripts.

To investigate the conditions under which the subset of protected transcripts could demonstrate an average two-fold increase in abundance in late torpor compared to abundance during an interbout arousal, we varied transcription and degradation rates in both temperature-independent and temperature-dependent manners. Specifically, we implemented the following mechanisms:

1. Higher rates of synthesis for protected transcripts resulting from different distributions of v_sr,w_.
2. Lower rates of degradation for protected transcripts resulting from different distributions of k_dr,w._
3. Protective, temperature dependent mechanisms for protected transcripts to reduce the degradation rate k_dr_ at low Tb to levels below those predicted by Q10 effects.

Theoretically, a fourth mechanism that protects rates of RNA synthesis in a temperature-dependent manner analogous to mechanism 3 could exist. However, it is biologically implausible because RNA polymerase II is required for synthesis of all transcripts and is temperature sensitive with a Q10 of 3 in ground squirrels (van Breukelen and Martin 2002). Moreover the high energy requirements of transcription are inconsistent with the high need for energy conservation during torpor.

Mechanism 1: Higher rates of synthesis. To increase the average transcription rate, v_sr,w_, of the protected population, we increased the log mean parameter mu of the associated v_sr,w_ distribution. Because steady state abundances for fixed Tb are determined by the ratio of v_sr_ and k_dr_, changes to the distribution of v_sr,w_ for the protected population would result in either a compensatory change in k_dr_ or a compensatory change in the steady state distribution of the abundance of protected transcripts in the warm animal. We separately considered cases in which the increase of mu resulted in i) an increased steady state abundance of the transcript during the interbout arousal or ii) a compensatory increase in the degradation rate constant to maintain steady state levels consistent with the bulk population. For case i, initial steady state values were maintained as the ratio of individual v_sr,w_ to k_dr,w_, resulting in an increase in average steady state value for the protected transcripts. In case ii, we redefined k_dr,w_ to be equal to v_sr,w_ divided by the average steady state abundance for the bulk transcripts. Therefore, in the protected population, increased transcription rates were balanced by increased degradation rates, but the distribution of steady states was unchanged.

Mechanism 2: Lower rates of degradation. To decrease the average degradation rate k_dr,w_ , of the protected population, we increased the log mean parameter mu of the associated distribution for the half-lives, t_1/2_. As in Mechanism 1, we separately considered cases in which this increase of mu resulted in: i) an increased steady state abundance of the transcript during interbout arousal, or ii) a compensatory decrease in the transcription rate, v_sr,w_, to maintain steady state levels consistent with the bulk population. Implementations of these compensations were analogous to the implementations described for Mechanism 1.

Mechanism 3: Temperature-dependent protection of degradation. To implement a mechanism such as sequestration that would prevent degradation of protected transcripts below certain temperatures, we replaced the temperature dependence specified by the Q10 effect on degradation rate, k_dr_, with a temperature-dependent degradation rate that is a fixed percentage of the warm degradation rate for temperatures below a temperature threshold:


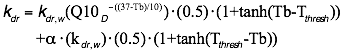


where T_thresh_ is the threshold temperature below which protection occurs and alpha is the percentage of the warm degradation rate that persists at the protected temperature. We considered threshold temperature values ranging from 5°C to 20°C. Note that since Q10=2 for degradation, the expected Q10 effect on degradation rate at 5°C is a scaling by 2 ^(-(37-5)/10)^ = 0.1088. Therefore, to represent a protective effect of the modeled mechanism beyond that predicted by Q10, we considered values of alpha less than 10%.

References

Burka, E. R. (1969). "Characteristics of RNA degradation in the erythroid cell." J Clin Invest **48**(7): 1266-1272. 10.1172/JCI106092

Schwanhausser, B., D. Busse, N. Li, G. Dittmar, J. Schuchhardt, J. Wolf, W. Chen and M. Selbach (2011). "Global quantification of mammalian gene expression control." Nature **473**(7347): 337-342. 10.1038/nature10098

van Breukelen, F. and S. L. Martin (2002). "Reversible depression of transcription during hibernation." J Comp Physiol B **172**(5): 355-361. 10.1007/s00360-002-0256-1
